# Multiple myeloma in Armenia during the period 2006–2018: facts and discussion

**DOI:** 10.1186/s12885-021-08676-w

**Published:** 2021-08-21

**Authors:** Narine Ghazaryan, Samvel Danelyan, Samvel Bardakhchyan, Anahit Saharyan, Lusine Sahakyan

**Affiliations:** 1 Armenian Haematology Center Aft.Prof. R.Yeolyan, Yerevan, Armenia; 2 Laboratory of Toxinology and Molecular Systematics, Institute of Physiology, Yerevan, Armenia; 3 Yerevan State Medical University after Mkhitar Heratsi, Yerevan, Armenia

**Keywords:** Multiple myeloma, Incidence, Survival, Armenia

## Abstract

**Background:**

It is known that one of the reasons for the increased incidence of hematological malignancies is caused by the growth of multiple myeloma (MM). Worldwide, approximately 159,985 new cases of MM are diagnosed representing 0.9% of all cancer diagnoses and 106,105 patients will die from MM accounting for 1.1% of all cancer deaths per year. Despite significant advances in the MM treatment the mortality rates are still high. The presented study is the first accurate epidemiological study of ММ in Armenia for the period of 2006–2018.

**Methods:**

The initial data for this retrospective cohort survey were derived from ambulance cards, hospitalization journals, and clinical data from the Registry of Blood Diseases at the Yeolyan Hematology Center.

**Results:**

Data analysis showed that during 2006–2018 the average annual incidence for the MM was 1.2 per 100,000 population. A significant increase was observed in 2018 compared to 2006, 1.9 vs. 0.7 per 100,000 population. Interestingly, there were no sex differences in the overall MM incidence during the study period.

According to the received data, during the period of the 2006–2009 and 2014–2018 the 1-year survival rate for both sexes decreased dramatically from 83 to 64.1% at age group 60 years and below and from 78.5 to 68.1% in group 60 years and over. The 1-year overall survival (OS) for both sexes decreased by 18.9% for patients (≤60 age group) and 10.4% (> 60 age group) in the period of 2006–2009 to the period of 2014–2018.

**Conclusions:**

The incidence rates for the MM increased during the analyzed period. Our study showed that males and females in the age group 60 years and below had better 5-year overall survival compared to elder ones and females have better survival.

## Background

The combat against hematological malignancies is primarily aimed at reducing prevalence, incidence, mortality and improving the quality of life of cancer patients through prevention, early diagnosis, effective therapy and palliative care. Among hematological malignancies, in terms of frequency, MM is the second, following lymphomas, and accounts for 1% of all neoplasias, 2% of all deaths from neoplasias and 12–15% of onco-hematological diseases [1–11].

The incidence and prevalence of MM increases with age [12]. The disease is more common in men than women. Several studies have shown that the age-related incidence of MM varies throughout the world․ The incidence is the highest in industrialized nations including North America, Australia/New Zealand, and Europe and the incidence appears to be rising in these regions while remaining relatively stable in Asian countries [1, 2, 13–20]. Age-standardized incidence and death rates are the highest in the Australasian/New Zealand, North American, Northern and Western European populations, and the lowest – in Asia and Western Africa [4, 18, 20–22]. Despite the fact that the incidence of MM has increased significantly in different parts of the world in the last 10–20 years, in some areas of the US and the UK it has reached its plateau [1, 12, 21–28]. The latter might be due to improvement in clinical diagnosis of cases.

To the best of our knowledge, there have been no studies of MM morbidity in the Republic of Armenia so far. In order to fill this gap, the aim of this study was to identify patterns and trends in the incidence of MM in the Republic of Armenia from 2006 to 2018.

## Methods

The Hematology Center of Armenia is the only medical institution in Armenia, which performs diagnostics and treatment of patients with MM. The main data sources for this retrospective cohort study were outpatient and dispensary cards, hospitalization journals, as well as clinical data from the Register of Blood Diseases of the Hematology Center after. Prof. R. Yeolyan. This information has been supplemented by the data from National Oncological Center after V.A. Fanarjyan as well as from death registration service at the Ministry of Justice RA. In addition, in Armenia, under the Ministry of Health, there is a program called e-health, to which all medical institutions in Armenia are connected, and any medical event with the patient is introduced from everywhere in this program. Even if the patient is not hospitalized, but simply visits the clinic for consultation or analysis, all this information is electronically stored in the register and available to the physician. The register program eliminates duplication of the records. Information about follow up was also taken from these sources. The analysis of the demographic data on MM incidence in Armenia was carried out for the period of 2006–2018 (Table 1). All patients diagnosed with MM in Armenia during this period were included in the study. Demographic indicators were derived from the database of the Statistical Committee of Armenia (https://www.armstat.am/en/).

**Table 1:** Characteristics of patients with multiple myeloma diagnosed between 2006 and 2018 in Armenia

Characteristics	Number of patients	(%)
**Total**	**502**	**(100)**
Sex		
Male	242	(48.2)
Female	260	(51.8)
Race and ethnicity Indo-European/Armenian	502	(100)
Age group		
25–34	3	(0.6)
35–44	22	(4.4)
45–54	103	(20.5)
55–64	200	(39.8)
65–74	127	(25.3)
75+	47	(9.4)

Figures are numbers (with percentages in total number of patients)

Data was analyzed with the IMB SPSS version 23 statistical software. We carried out descriptive statistical analysis and presented all the continuous variables by means, medians, and standard deviations (SD). We compared overall survival of patients below and above 60 years. Also, overall survival was analyzed by the year of diagnosis and the patients’ cohort was subdivided into three groups: diagnosed during 2006–2009, 2010–2013, 2014–2018. The Kaplan Meier method of survival analysis was used to display the overall survival and log-rank tests used to assess the significance between the survival rate among the males and females diagnosed between 2006 and 2018. Differences were considered significant if the *P*-value was < 0.05. The endpoint event was defined as the five-year overall survival measured with the 95% confidence level, where the survival time variable was defined as the time between diagnosis and death, and censoring date for the alive patients was taken as the 31st of March 2019. Patients who were lost from follow up were excluded from the final analysis.

Diagnosis of MM and the definition of the stage were carried out according to the criteria proposed by the International Myeloma Working Group [7]. The patients received melphalan and prednisolone (MP), cyclophosphamide and prednisolone (CP), vincristine, doxorubicin, and dexamethasone (VAD) and also according to new protocols other treatment such as (Cyclophosphamide, bortezomib, Doxorubicin and Dexamethasone (CVDD) [1, 21, 29]. The response to the therapy was assessed according to the criteria of the European Group for Blood and Marrow Transplant (EBMT) guidebook. Overall survival was determined by the period between the date of diagnosis and the date of death. The direct method of adjustment was used for the elimination of possible influence of changes in the age structure of population for a period of 10 years. The time series data were analyzed using the least squares regression analysis.

## Results

**Fig. 1:**
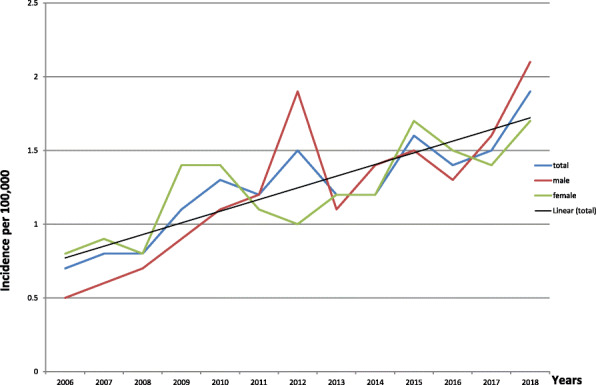
The incidence rates of MM in Armenia for the period 2006–2018

The demographic characteristics of the patients are presented in Table 1. Totally 502 patients during the mentioned period were enrolled in the study. We had follow up information for 483 of them. Median follow up time was 2.5 years (range 0–11.5 years). All the 502 patients in our study were Indo-European/Armenians and 75% of the patients were older than 55 years (Table 1). We found that the average annual incidence was 1.2 per 100,000 population during 2006–2018 period. The average annual gender-specific incidence rates of MM in Armenia are presented in Fig. 1. The analysis of the annual average incidence rates of MM revealed a tendency to a certain increase in the incidence rate in the study period both for males and females. The linear analysis was conducted in relation to the incidence rates in general.

After a direct adjustment MM rates still demonstrated a significant increase in 2018 compared to 2006, 1.9 vs. 0.7 per 100,000 population. In some cases, a higher incidence rate was registered among the male than female, for instance in 2012 it was 1.9 and 1.0 respectively, in some cases among the female than male (1.4 vs 0.9 in 2009). Interestingly, there were no sex differences in the overall MM incidence during the study period (Fig. 1).

**Fig. 2:**
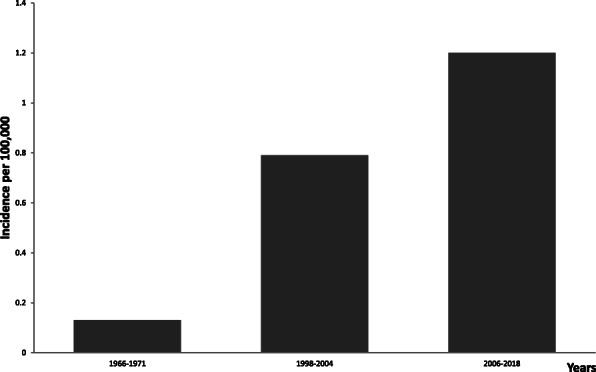
Comparison of incidence rates of MM for 2006–2018, 1998–2004, and 1966–1971

We observed a significant increase in the incidence of MM during the three investigated periods as well. The average annual incidence rates of MM (Fig. 2) increased in the last 12 years compared to those reported by early studies (1966–1971 and 1998–2004) [30]. A number of studies have reported increasing MM incidence over time in the world as well [2, 4, 9, 11, 21, 23].

**Fig. 3:**
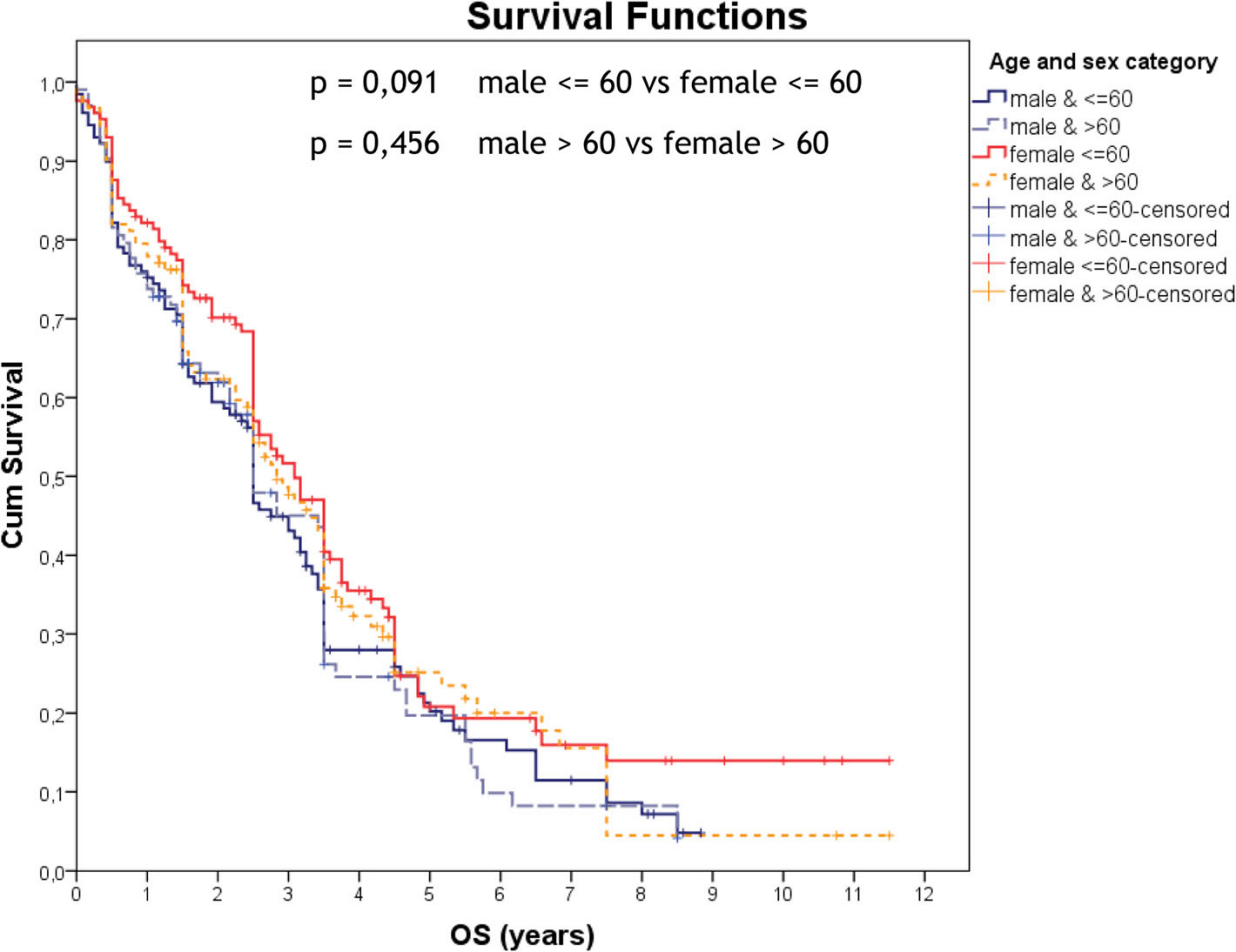
Kaplan-Meier curves according to the age and sex of the patients

In addition, we analyzed the overall survival parameters of 1- and 5-year of patients with MM for the 2006–2009, 2010–2013, 2014–2018 period (Table 2). According to the received data, during the period of the 2006–2009 and 2014–2018 the 1-year survival rate for both sexes decreased dramatically from 83 to 64.1% at age group 60 years and below and from 78.5 to 68.1% in the group 60 years and over. The 1-year overall survival for both sexes decreased by 18.9% for patients (≤60 age group) and 10.4% (> 60 age group) in the period of 2006–2009 to the period of 2014–2018. But when we looked at 5-year survival rates the overall survival for both sexes were even lower 18.5% (≤60 age group) and 17.6% (> 60 age group) for the 2010–2013 period. The 5-year overall survival for females was 23.4% (≤60 age group) and 22.2% (> 60 age group) for the 2010–2013 period which was higher compared to the male 13.6% (≤60 age group) and 13.1% (> 60 age group) (Fig. 3, Table 2).

**Table 2:** Overall survival for the 2006–2009, 2010–2013, 2014–2018 period of follow-up for patients with multiple myeloma in Armenia (1- and 5-year estimates with 95% confidence level)

		1-year survival rates						5-year survival rates					
		Total		Male		Female		Total		Male		Female	
		≤60	> 60	≤60	> 60	≤60	> 60	≤60	> 60	≤60	> 60	≤60	> 60
2006–2009	%	83	78.5*	87.5	95*	80	72.2*	21.3	17	17.6	11.1*	25	22.9*
	CI	73.2–92.8	66.6–76.5	74.3–100	84.9–100	66.1–93.9	55.6–88.8	10.6–32	6.1–27.9	2.4–32.8	0–25.6	10–40	7.3–38.5
	n	56	46	24	18	32	28	56	46	24	18	32	28
2010–2013	%	85.5	82.1	75.8	87.6*	87.5	84.2	18.5	17.6*	13.6	13.1	23.4	22.2*
	CI	78.5–92.5	76.6–91.6	63.8–87.8	76.7–98.5	78.2–96.8	70.4–98	10.8–26.2	8.1–27.1	4–23.2	1.9–24.3	11.5–35.3	6.5–37.9
	n	98	62	49	35	49	27	98	62	49	35	49	27
2014–2018	%	64.1	68.1	48.9	66.9*	73.8	72	–	–	–	–	–	–
	CI	54.9–73.3	59.7–76.5	35.4–62.4	54.6–79.2	61.7–85.9	60.7–83.3						
	n	104	117	53	56	51	61						

*р **< 0.05; Comparison of age ≤ 60 and > 60**

## Discussion

During the study period (2006–2018), 502 primary MM patients were registered and / or treated at the Hematological Center of Armenia. The median age was 60.6 years (range 31–89) with 260 (51.8%) female and 242 (48.2%) male patients.

It is noteworthy that the incidence of MM overall was similar in females and males 1.23 to 1.22 respectively during the study period in Armenia which is very interesting as males are more commonly affected with MM than females in the world. We do not think that women get sick more often than men in Armenia, probably women seek medical help more which could be the reason for the higher detectability. The MM incidence rate increased with the age in most of the age groups, which was consistent with the previous study in Armenia [30].

First, a possible explanation that we have an increase in the incidence could be improvements in MM diagnosis in Armenia. This upward trend of incidence might be explained partly by some factors such as aging and excess body weight [15], but much of this trend was largely unexplained and the further study focused on the etiology of MM. Furthermore, other factors such as adverse environmental conditions could potentially increase the risk of myeloma [19]. Our data are consistent with the findings from other countries [1, 2, 6, 23].

With the implementation of new treatment agents, survival rates for MM have increased dramatically during recent years in developed countries: for example, 5-year overall survival rate in US or Germany are now exceeding 50% [31], is approximately 44% in Canada [32], 49% in Moscow, Russia [33]. The situation is somehow different in developing countries where not all novel drugs are available: the study in two centers of Iran reported 5-year overall survival for MM to be 35.6% [34], while in the Niger delta, Nigeria this value is dramatically less than - 7.6% [35].

Since Armenia is a developing country, the 5-year overall survival is not as high as in developed countries. The 5-year overall survival for both sexes was 18.5% (≤60 age group) and 17.6% (> 60 age group) for the 2010–2013 period in Armenia, which is much lower compared to the developed world. This can be due to late admission, poor affordability of new treatment drugs (IMIDs, PIs, monoclonal antibodies), absence of autotransplantation in Armenia during the study period, and also insufficient coverage of medication expenses by the government.

## Conclusion

We observed that the incidence rates for the MM increased during the analyzed period. Moreover, our study showed that males and females in the age group 60 years and below had better 5-year overall survival compared to elder ones and females have better survival. Based on the disparities in MM burden, different strategies for disease prevention and control should be employed when the health policy is developed in the future.

## Data Availability

Datasets for the current study are not publicly available to protect the anonymity of the respondents.

## Abbreviations

MM: Multiple myeloma
MP: Melphalane and prednisolone
CP: Cyclophosphamide and prednisolone
VAD: Vincristine, doxorubicin, and dexamethasone
EBMT: European group for blood and marrow
